# Immune responses induced by a combined vaccination with a recombinant chimera of *Mycoplasma hyopneumoniae* antigens and capsid virus-like particles of porcine circovirus type 2

**DOI:** 10.1186/s12917-020-02560-8

**Published:** 2020-09-16

**Authors:** Yu Tao, Rui Yang, Jianhong Shu, Wenqian Zheng, Jian Chen, Yuehong Wu, Yulong He

**Affiliations:** Department of Biochemistry and Molecular Biology, College of Life Sciences and Medicine, Zhejiang Sci-Tech University, 928 Second Avenue, Xiasha Higher Education Zone, Hangzhou, 310018 China

**Keywords:** Combination vaccine, *Mycoplasma hyopneumoniae*, Porcine circovirus type 2, Recombinant chimera, Virus-like particles

## Abstract

**Background:**

*Mycoplasma hyopneumoniae* (Mhp) and porcine circovirus type 2 (PCV2) are two important pathogens causing Mycoplasma pneumonia of swine (MPS) and porcine circovirus diseases and porcine circovirus-associated diseases (PCVDs/PCVADs), respectively, and resulted in considerable economic loss to the swine industry worldwide. Currently, vaccination is one of the main measures to control these two diseases; however, there are few combination vaccines that can prevent these two diseases. To determine the effect of combination immunization, we developed capsid-derived (Cap) virus-like particles (VLPs) of PCV2 and a new recombinant chimera composed of the P97R1, P46, and P42 antigens of Mhp. Then we investigated the immune responses induced by the immunization with this combination vaccine in mice and piglets.

**Results:**

The high level antibodies against three protein antigens (P97R1, P46, and P42 of Mhp) were produced after immunization, up to or higher than 1:400,000; the antibody levels in Pro group continuously increased throughout the 42 days for all the antigens tested. The lymphocyte proliferative response in PCV2 group was stronger than that in PBS, VP, Mhp CV in mice. The antibody levels for Cap remained stable and reached the peak at 35 DAI. The IFN-γ and IL-4 in sera were significantly enhanced in the Pro group than that in the negative control-VP group on Day 14 and 28 post-the first immunization in piglets.

**Conclusions:**

Above all, the combination immunization could induce humoral and cellular immune responses against all four antigens in mice and piglets. Therefore, our approach is a simple and effective vaccination strategy to protect pigs against MPS and PCVD/PCVAD.

## Background

Mycoplasma pneumonia of swine (MPS), also known as porcine enzootic pneumonia (PEP) or enzootic pneumonia (EP), is mainly caused by the pathogen *Mycoplasma hyopneumoniae* (Mhp). This chronic respiratory disease is widely spread all over the world and causes considerable economic loss [1, 2]. The specific adhesion of Mhp with cilia of epithelial cells of the respiratory tract is the key step for Mhp infection. Multiple functional adhesion factors (e.g. P97, P46, P42, P216, P102, P95, and P159), found in recent years, are involved in the adhesion process and are tightly associated with the pathogenicity and immunogenicity of Mhp [3–8]. The C-terminal region of the adhesin P97 (P97R1), which are much conserved among the different strains of Mhp, plays an important role in the adherence [9–11]. Many researchers have focused on studying this conserved region The molecular chaperone DnaK (P42) and membrane surface protein (P46) are another two important adhesion factors, which can be potentially used in vaccine research [4, 5, 12–15].

Porcine circovirus type 2 (PCV2) causes porcine circovirus diseases and porcine circovirus-associated diseases (PCVDs/PCVADs), which present clinically as postweaning multisystemic wasting syndrome (PMWS), porcine dermatitis nephritic syndrome (PDNS), reproductive disturbance, and enteritis [16–18]. The capsid (Cap) protein encoded by ORF2 is the major immunogen and plays critical role in the diagnosis and vaccine development of porcine circovirus.

Virus-like particles (VLPs) are hollow particles without viral genetic material, which are recombinantly expressed and assembled by capsid proteins of a virus in heterologous systems. VLPs have similar morphology, antigenicity and immunogenicity to real virus particles but without infectivity, so they are one promising candidate vaccine against infectious diseases [19, 20].

Previous studies have reported that PCV2 has coinfection with other swine pathogens, such as Mhp, porcine parvovirus and swine influenza. This coinfection may cause immunosuppression to reduce the host immunity and eventually increase mortality because of increased risk of infection [21]. Currently, mainly separate vaccination is relied on for each disease, which causes great inconvenience to the immune process and causes an economic burden to farm managers. It has been reported that a chimera composed of the P97R1, P95, P46 and P42 antigens of Mhp showed good results in mouse immune responses [4]. Moreover, Cap protein of PCV2 can be assembled into VLPs by the *Escherichia coli* (*E. coli*) system and were able to induce specific antibody responses in pigs [22]. Therefore, the combined injection of several antigens of Mhp and Cap antigen of PCV2 may achieve simultaneous protection on both diseases. In the present study, we first developed a recombinant chimeric protein composed of the P97R1, P46, and P42 antigens of Mhp and recombinant Cap VLPs of PCV2 and then evaluated the immunogenicity of a combined vaccination using these two kinds of proteins in mice and piglets.

## Results

### Vector construction and generation of the recombinant proteins

**Fig. 1:**
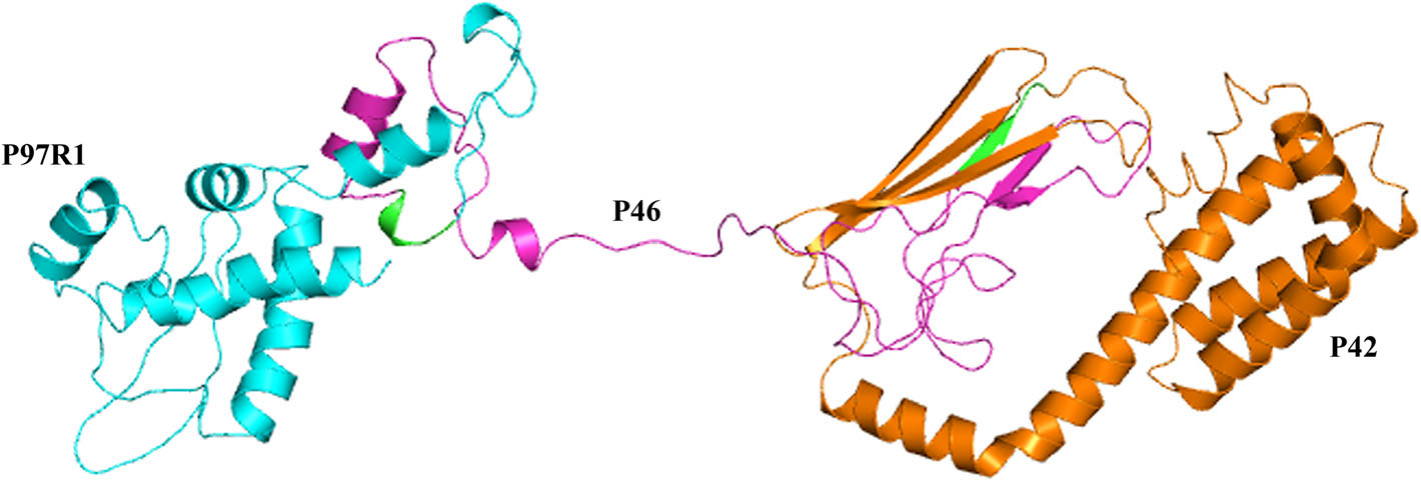
Three-dimensional structure prediction of the chimeric protein rP97R1P46P42

The characteristics of the antigenic sequences used in this study are summarized in Table 1. The predicted three-dimensional structure of the chimeric protein rP97R1P46P42 is shown in Fig. 1. The blue, purple and orange parts represent the P97R1, P46 and P42 proteins, respectively. The green parts represent two GGSG flexible linkers between P97R1 and P46 and between P46 and P42.

**Table 1:** Characteristics of the antigens selected in the recombinant protein

Antigens	Protein name	NCBI accession number	Features/function	Original amino acid length (aa)	Selected fragment (aa)
Mhp	P97R1	ADQ90328.1	Cilium adhesin	1082	788–915
	P46	ADQ90718.1	46 kDa surface antigen	120	24–120
	P42	ADQ90292.1	Chaperone protein DnaK	622	434–600
PCV2	Cap	ARW74078.1	Capsid protein	234	42–234

**Fig. 2:**
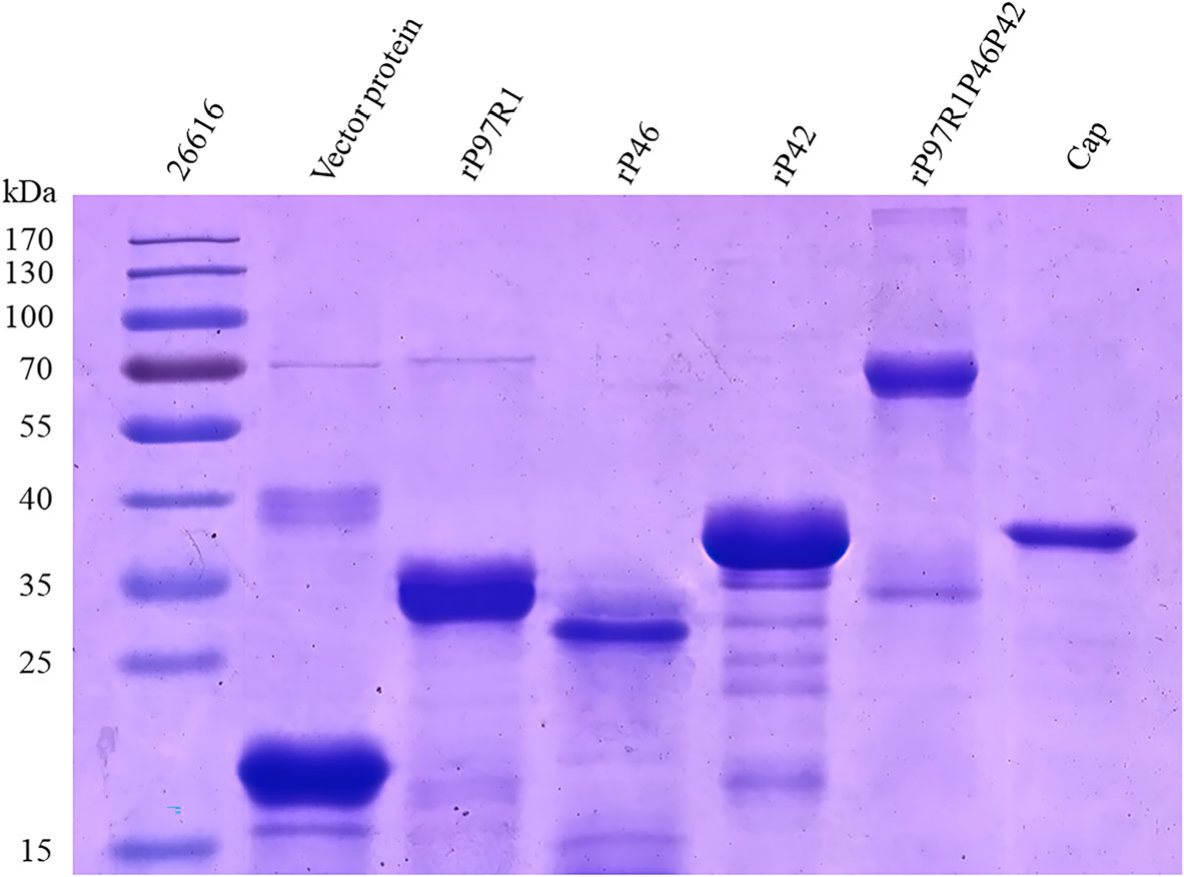
Identification of proteins by SDS-PAGE. The lanes left-to-right are 26,616 prestained protein marker (Thermo Fisher, USA), pET-32a expressed proteinrP97R1, rP46, rP42, rP97R1P46P42, and Cap

**Fig. 3:**
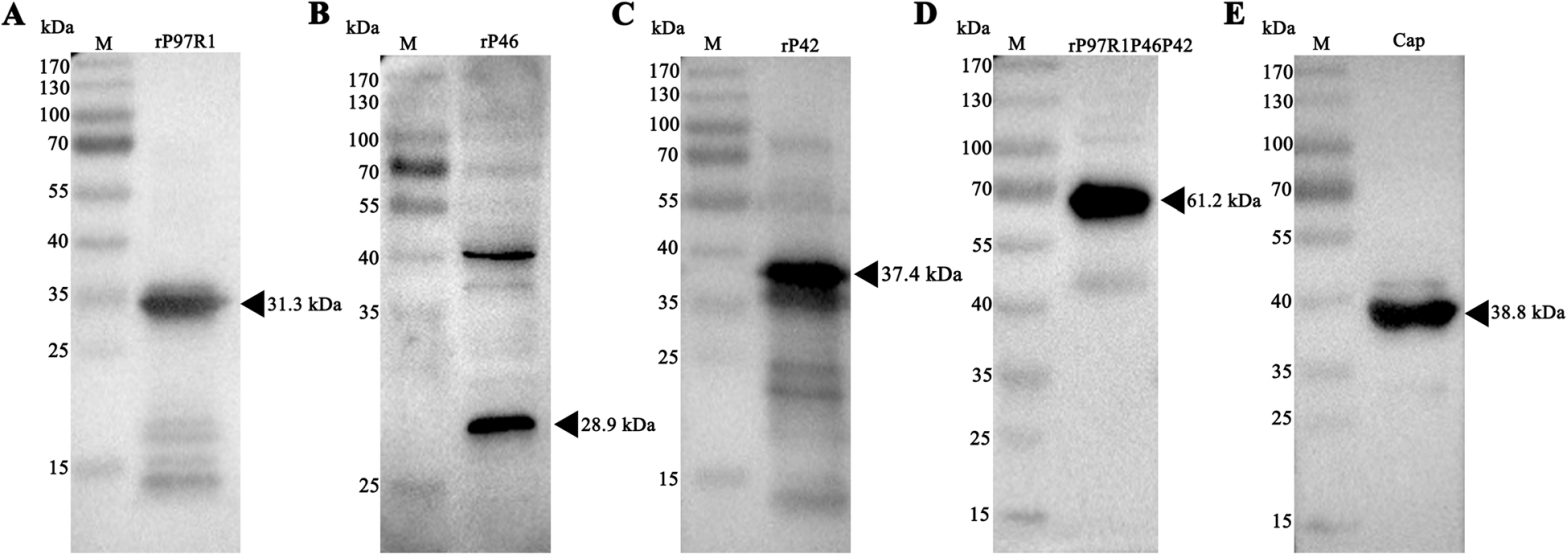
Identification of proteins by Western blotting. Western blotting analysis with a monoclonal anti-His antibody, showed that the molecular masses of rP97R1 (**a**), rP46 (**b**), rP42 (**c**), rP97R1P46P42 (**d**), and Cap (**e**) were approximately 31.3 kDa, 28.9 kDa, 37.4 kDa, 61.2 kDa, and 38.8 kDa, respectively

The sequences of the recombinant plasmids did not have unwanted mutations (data not shown). The purity of the pET32a vector, rP97R1, rP46, rP42, rP97R1P46P42, and capsid (Cap) proteins were analyzed by SDS-PAGE (Fig. 2) and Western blotting (Fig. 3, the full-length blots are presented in Supplementary Fig. (1, 2, 3, 4 and 5) for A to E respectively, the cropped area of the picture was marked with a red box line). It demonstrated that the expression and purification of recombinant proteins were in line with our expectations, and these values corresponded to the expected molecular weights of pET32a vector protein (20.4 kDa), rP97R1 (31.3 kDa), rP46 (28.9 kDa), rP42 (37.4 kDa), rP97R1P46P42 (61.2 kDa), and Cap (38.8 kDa).

### Assembly of cap VLPs

**Fig. 4:**
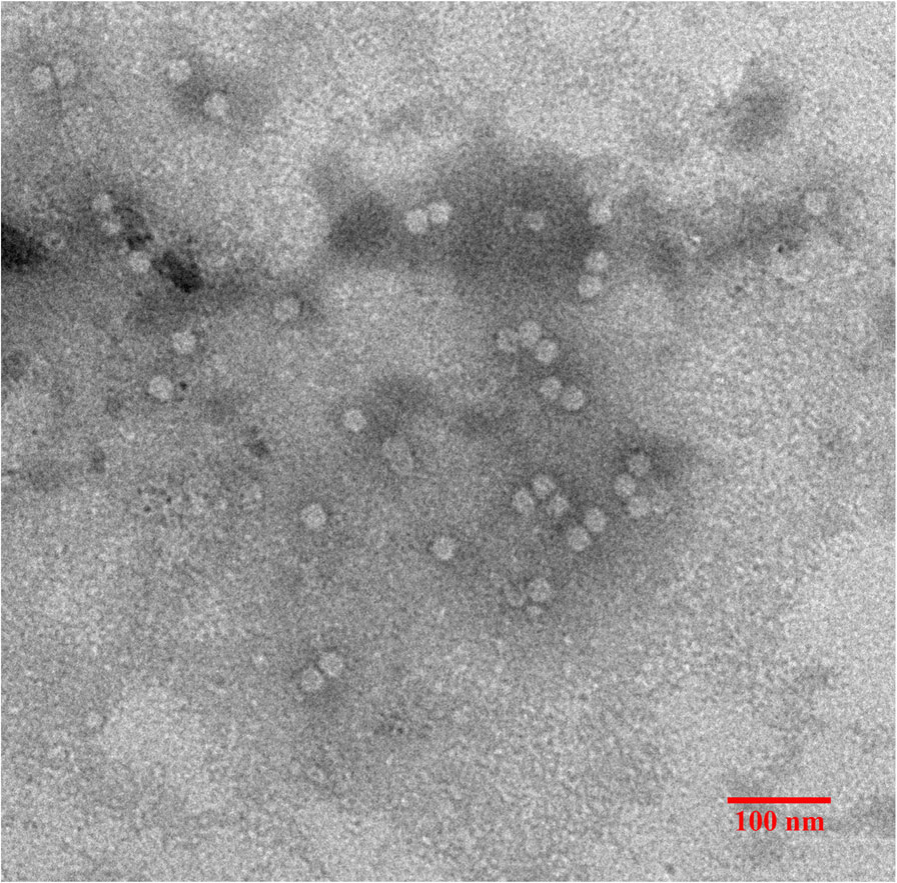
TEM images of the Cap protein VLPs of PCV2

The soluble expressed Cap proteins were purified by ultracentrifugation to detect whether the nuclear localization signal-deleted Cap protein was able to self-assemble into VLPs in *E.coli*. TEM images revealed that Cap proteins could assemble into VLPs with diameters ranging from 15 to 20 nm (Fig. 4).

### Titers of antibodies and antigenicity of the recombinant proteins

**Fig. 5:**
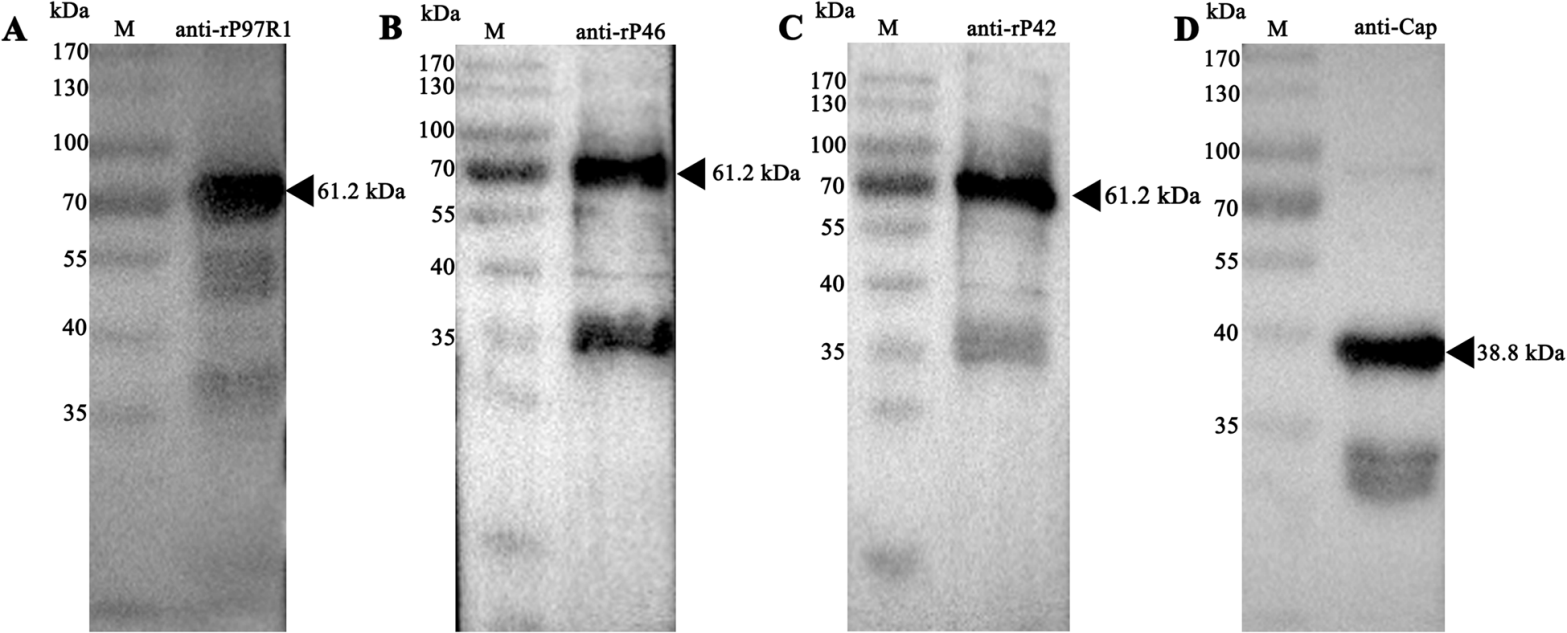
Antigenic analysis of the rP97R1P46P42 chimera and Cap VLPs. The chimeric protein rP97R1P46P42 was specifically recognized by polyclonal (**a**) anti-rP97R1 (1:2000), (**b**) anti-rP46 (1:1000) and (**c**) anti-rP42 (diluted 1:2000) antibodies. The Cap VLPs were specifically recognized by a polyclonal (**d**) anti-Cap antibody (1:5000)

Titers of Protein A column-purified polyclonal antibodies were 1:409600 (anti-rP97R1), 1:204800 (anti-rP46), and 1:409600 (anti-rP42), as determined by indirect ELISA (Table 2). To verify whether the three polyclonal antibodies were specific and if the components of the combined vaccination maintained the original antigenic epitopes, a Western blotting analysis was performed. The rP97R1P46P42 chimera and Cap VLPs were recognized by polyclonal anti-rP97R1, anti-rP46, and anti-rP42 antibodies, and anti-Cap antibody reacted against each subunit (Fig. 5, the full-length blots are presented in Supplementary Fig. (6, 7, 8 and 9) for A to D respectively, the cropped area of the picture was marked with a red box line). The pET32a expressed protein could not be recognized by the four polyclonal antibodies.

**Table 2:** Determination of three polyclonal antibody titers

Antigens	Negative control	1:51200	1:102400	1:204800	1:409600	1:819200
rP97R1	0.129 ± 0.025	1.803 ± 0.162	1.070 ± 0.125	0.699 ± 0.063	0.428 ± 0.003	0.233 ± 0.008
rP46	0.117 ± 0.011	0.845 ± 0.164	0.572 ± 0.076	0.342 ± 0.049	0.217 ± 0.017	0.119 ± 0.015
rP42	0.143 ± 0.010	1.686 ± 0.069	1.156 ± 0.064	0.743 ± 0.058	0.416 ± 0.072	0.236 ± 0.035

### Cellular immune responses in mice

**Fig. 6:**
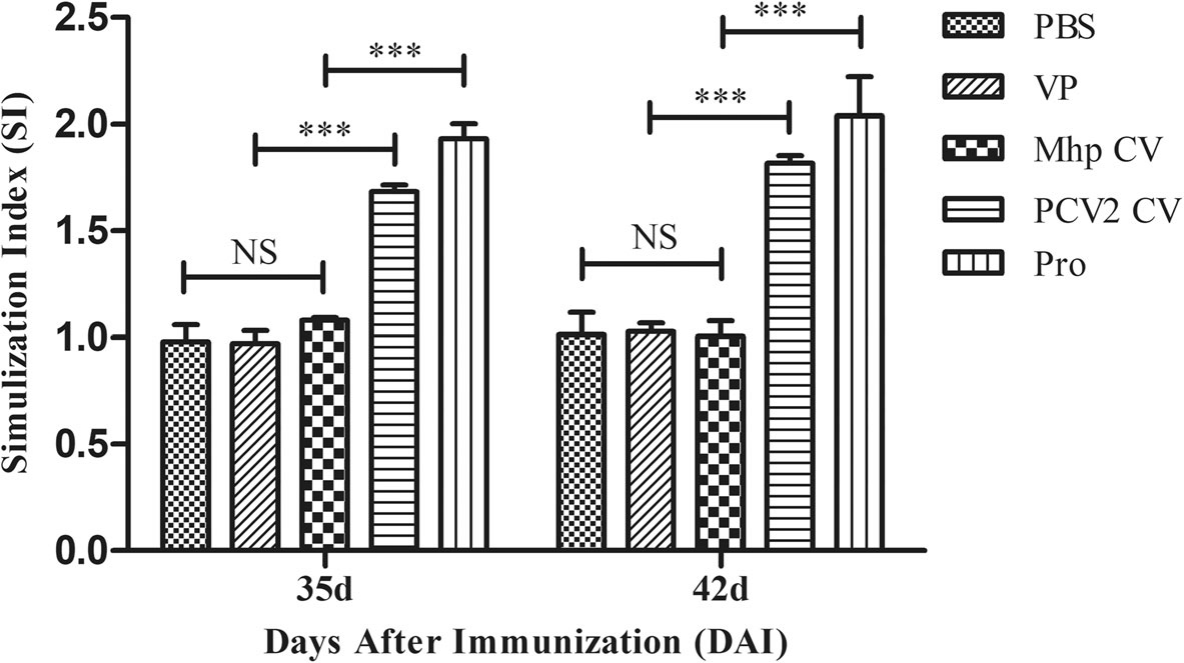
Lymphocyte proliferative responses in mice immunized with different vaccines. Numbers represent the stimulation index (SI) of lymphocyte samples collected at 35 DAI and 42 DAI. Samples were assayed in triplicate. Data represent the mean ± SD (*n* = 3). NS, no significant difference; ***P < 0.001, significantly different (Bonferroni test)

To investigate whether the combined vaccination could induce cell-mediated immune responses, the lymphocyte proliferative responses were examined at 35 and 42 DAI (Fig. 6). The stimulation index (SI) value was significantly higher in the Pro group than in the PBS, VP, Mhp CV and PCV2 CV groups. The difference was statistically significant (*P* < 0.001). The SI value from the PCV2 CV group was significantly higher (P < 0.001) than those from the PBS, VP and Mhp CV groups. The Concanavalin A control (positive control of lymphocyte proliferation assay) worked well (data not shown).

### Systemic humoral immune response in mice

**Fig. 7:**
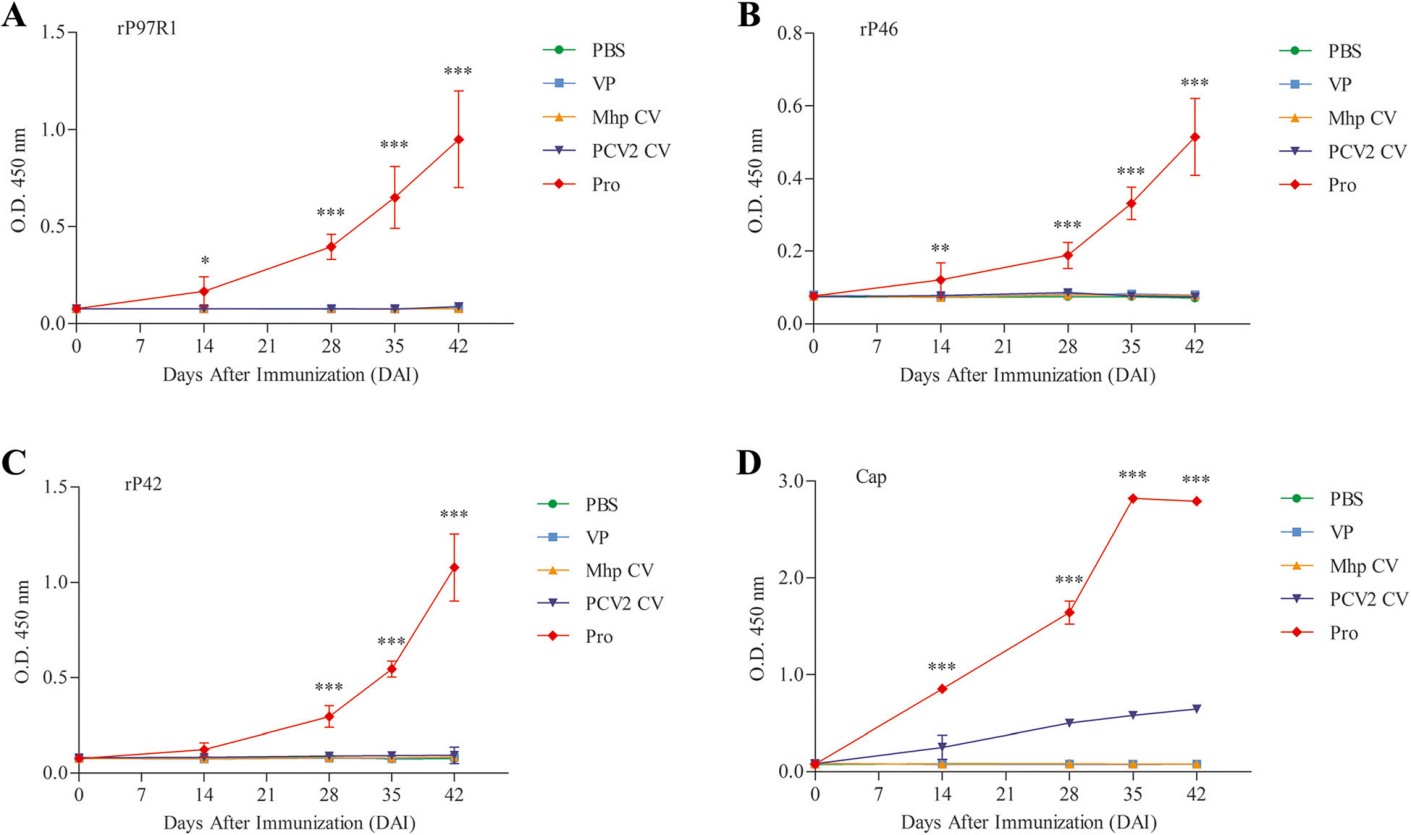
Analysis of IgG response induced by four recombinant proteins post immunization determined by indirect ELISA (**a**–**d**). **a**. Total IgG level against rP97R1. **b**. Total IgG level against rP46. **c**. Total IgG level against rP42. **d**. Total IgG level against Cap. Numbers represent the mean optical density at 450 nm (OD 450) of serum samples collected at 0, 14, 28, 35 and 42 DAI in each group. All analyses were performed in triplicate, and the error bars demonstrate standard deviations (SDs). Statistical significance was determined by two-way ANOVA. *P < 0.05, **P < 0.01 and ***P < 0.001, significantly different from the PBS, VP, Mhp CV and PCV2 CV groups (Bonferroni test)

The level of IgG and IgG isotype, specific for each antigen, were determined by indirect ELISA. The Pro group induced an antibody response that was significantly higher (*P* < 0.001) at 28 DAI than those in the other groups (PBS, VP, Mhp CV and PCV2 CV groups), and the antibody levels continuously increased throughout the 42 days for all the antigens tested (Fig. 7). The antibody levels for Cap remained stable and reached the peak at 35 DAI. The sera from the PBS, VP and Mhp groups did not induce the detectable specific antibodies.

**Fig. 8:**
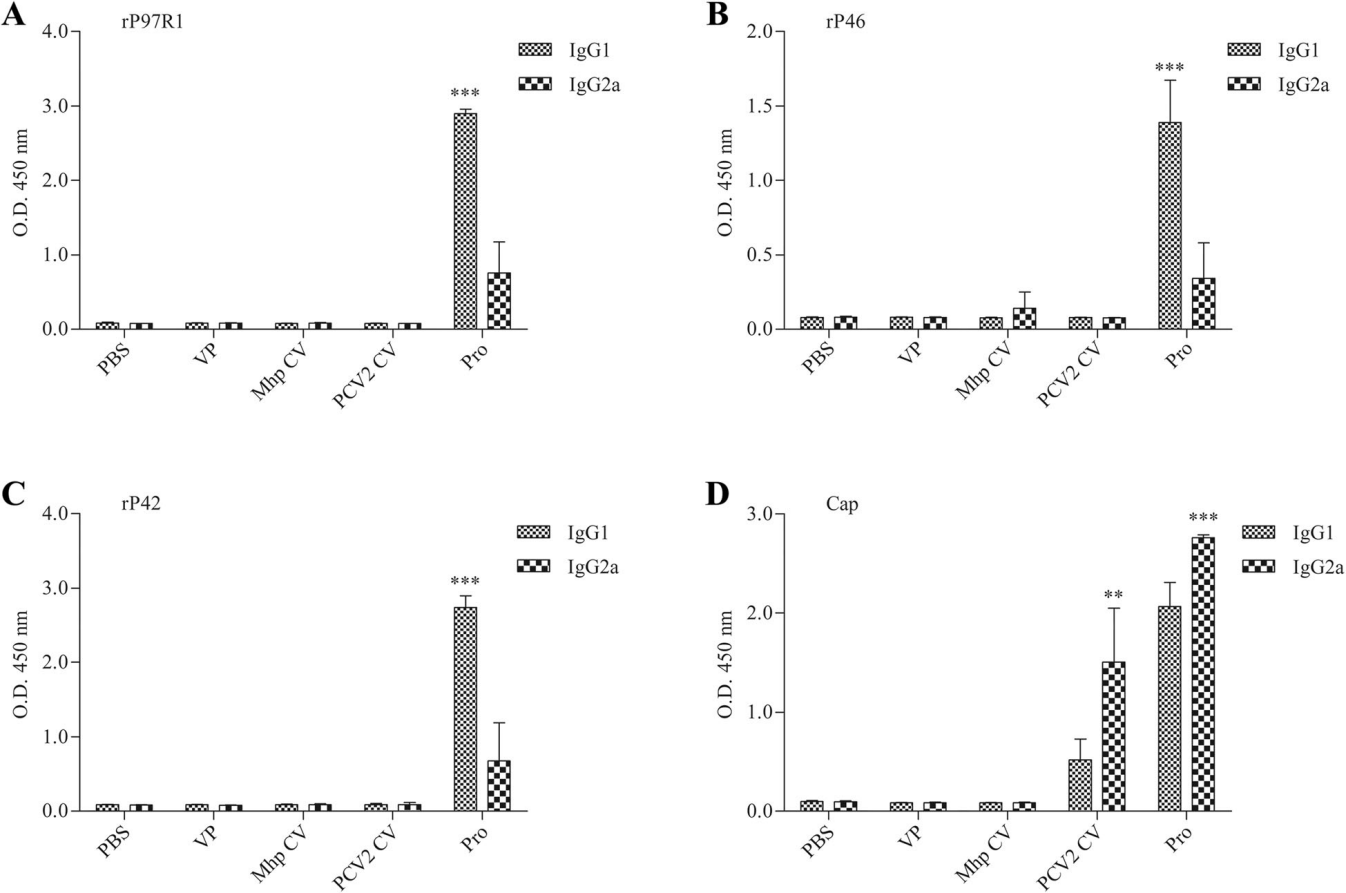
Analysis of IgG1 and IgG2a antibodies induced by immunization determined by ELISA with recombinant proteins in mice (**a**–**d**). Numbers represent the mean optical density at 450 nm (OD 450) of serum samples collected at 42 DAI in each group. All analyses were performed in triplicate, and error bars demonstrate standard deviations (SDs). Statistical significance was determined by two-way ANOVA. **P < 0.01 and ***P < 0.001 indicate significant differences between isotypes in each group (Bonferroni test)

Analysis of IgG isotypes showed that the Pro group presented the highest seroconversion for both isotypes against all the antigens (Fig. 8). The humoral immune response (IgG1) was predominant, (*P* < 0.001) against the three recombinant subunit antigens (rP97R1, rP46 and rP42) (Fig. 8a-c). The cellular immune response induced in the Pro group and the PCV2 CV group was predominant for only the recombinant Cap antigen (Fig. 8d). Sera from the PBS, VP and Mhp CV groups did not induce the detectable specific antibodies (Fig. 8a-d).

### Reactivity with field strains in mice

**Fig. 9:**
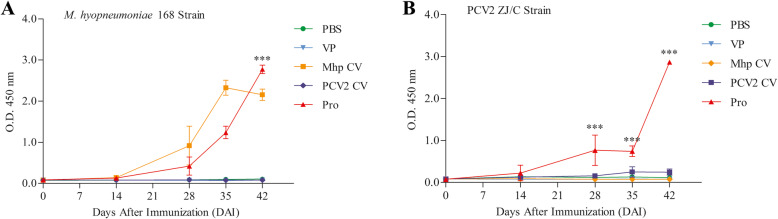
ELISA using Mhp and PCV2 extract as antigens. **a**. Total IgG level against Mhp 168 strain. **b**. Total IgG level against PCV2 ZJ/C strain. Numbers represent the mean optical density at 450 nm (OD 450) of serum samples collected at 0, 14, 28, 35 and 42 DAI in each group. All analyses were performed in triplicate, and the error bars demonstrate standard deviations (SDs). Statistical significance was determined by two-way ANOVA. ***P < 0.001, significantly different from the PBS, VP, Mhp CV and PCV2 CV groups (Bonferroni test)

ELISAs with Mhp strain 168 and PCV2 strain ZJ/C were performed with the sera from immunized mice. The levels of antibodies against Mhp strain 168 were remarkably higher (*P* < 0.001) in serum from the Pro group than in those from the other groups at 42 DAI (Fig. 9a). The serum from the Pro group had significantly higher (P < 0.001) levels of antibodies against PCV2 strain ZJ/C than those from the PBS, VP, Mhp CV and PCV2 CV groups, at 28, 35, and 42 DAI (Fig. 9b). The antibody levels against both Mhp strain 168 and PCV2 strain ZJ/C continuously increased after immunization.

### Humoral and cellular immune responses in piglets

**Fig. 10:**
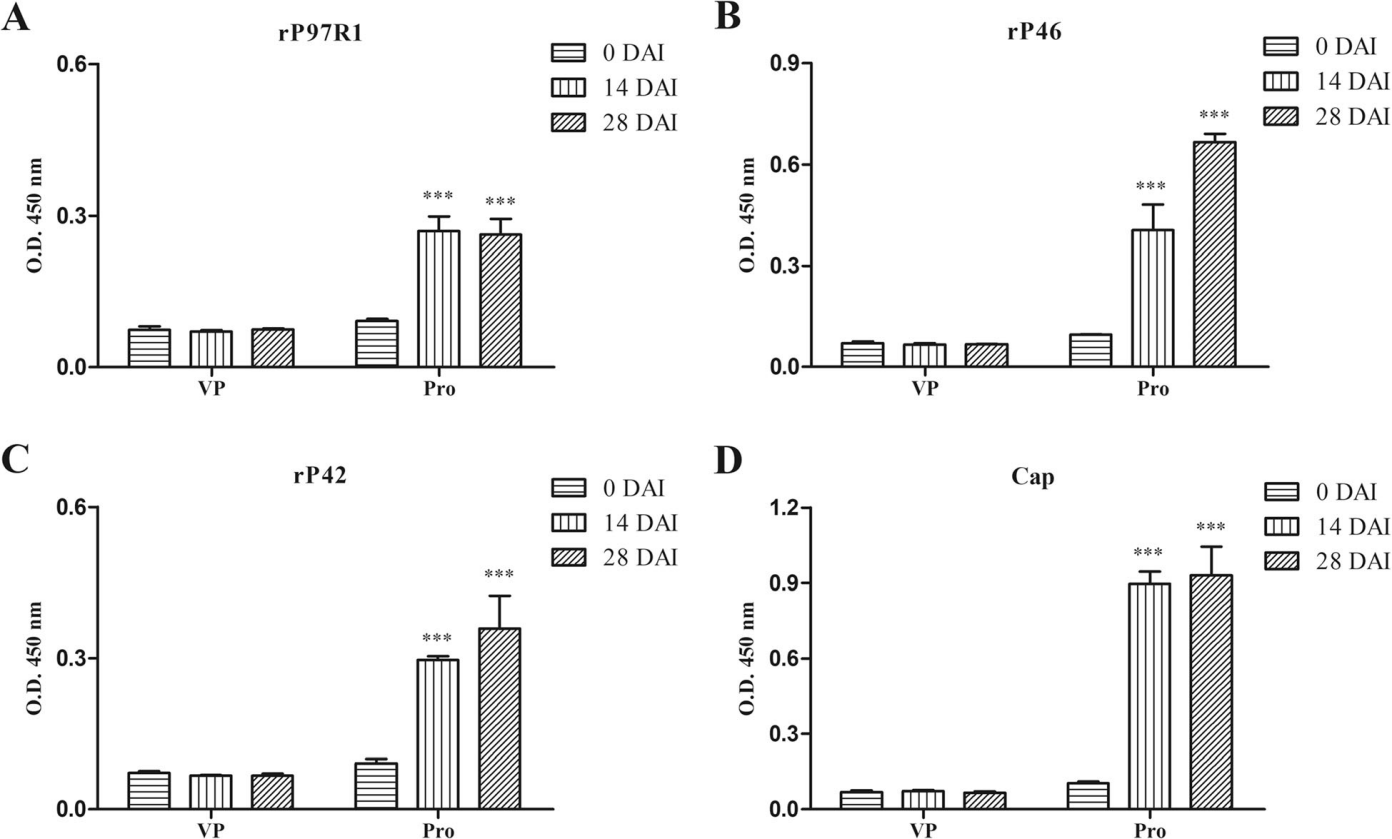
Analysis of IgG response induced by pig immunization determined by indirect ELISA with four recombinant proteins (**a**–**d**). **a**. Total IgG level against rP97R1. **b**. Total IgG level against rP46. **c**. Total IgG level against rP42. **d**. Total IgG level against Cap. Numbers represent the mean optical density at 450 nm (OD 450) of serum samples collected at 0, 14 and 28 DAI in each group. All analyses were performed in triplicate, and the error bars demonstrate standard deviations (SDs). Statistical significance was determined by two-way ANOVA. ***P < 0.001, significantly different from the VP group (Bonferroni test)

The humoral immune response induced in piglets was analyzed based on total IgG levels. The Pro group induced an antibody response that was statistically higher (*P* < 0.001) than that in the VP group at 28 DAI. The antibody levels from the serum of the Pro group continuously increased over 28 days for all the antigens tested (Fig. 10).

**Fig. 11:**
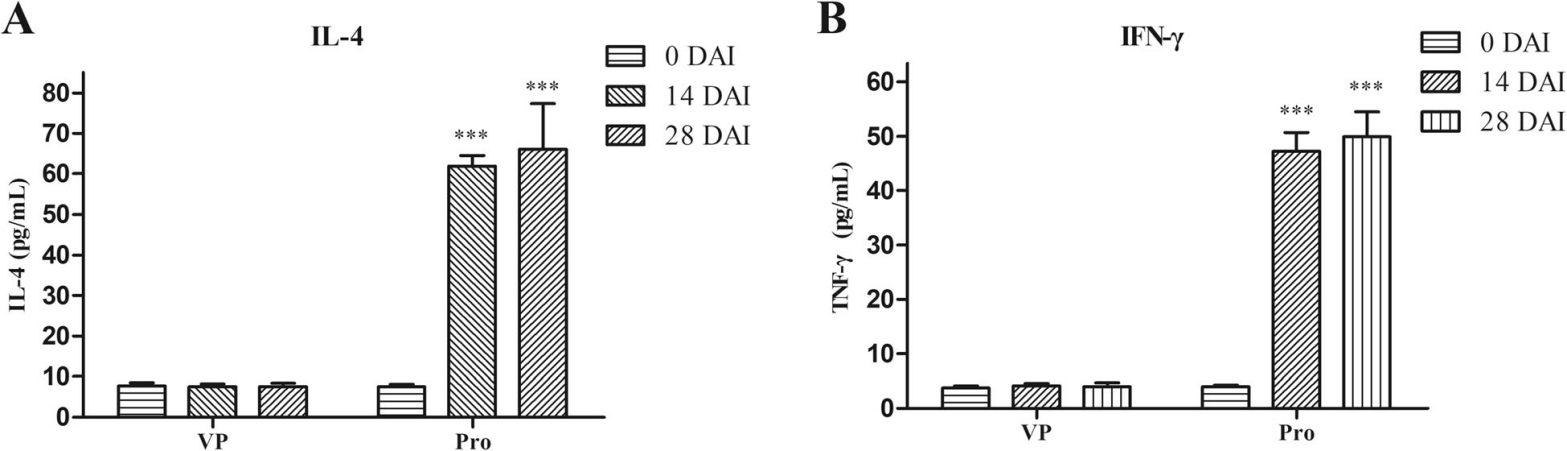
Analysis of IL-4 and IFN-γ levels in serum after pig immunization determined by indirect ELISA. **a** Concentration of the cytokine IL-4. **b** Concentration of the cytokine IFN-γ. Numbers represent the mean cytokine concentration in serum samples collected at 0, 14 and 28 DAI in each group. All analyses were performed in triplicate, and the error bars demonstrate standard deviations (SDs). ***P < 0.001, significantly different from the VP group (Bonferroni test)

The cellular immune response induced in piglets was analyzed by detecting the IFN-γ and IL-4 cytokines in serum. Significantly higher levels of IFN-γ and IL-4 were present in the serum of the Pro group than that in the negative control-VP group at 14 and 28 DAI, varying from 47.21 to 55.23 pg/mL and 52.94 to 72.79 pg/mL (P < 0.001), respectively (Fig. 11).

## Discussion

Currently, the main vaccines for MPS and PCVD/PCVAD are inactivated vaccines and attenuated vaccines. However, some studies have shown that these commercial vaccines for MPS could not induce satisfied levels of antibodies against Mhp antigens [23]. Therefore, there may still be some defects in commercial vaccines, although these commercial vaccines were prevalent used in swine industry. Because the large variety of adhesion factor proteins in Mhp causes difficulty in antigen selection and the development of protein vaccines involves problems of expression, assembly and purification, there are no genetically engineered vaccines for Mhp but a few genetically engineered vaccines for PCV2 available on the market now [24, 25]. Therefore, it is of great value to the development of genetically engineered vaccines for Mhp and PCV2.

The development of combined vaccines or combined vaccination strategies has gradually become the main trend to adapt to the needs of the market. Recently, researchers evaluated some of these combined vaccines and obtained good results in the immune reaction induced by them in mice or pigs [26–30]. Combined vaccines can not only reduce cross-infection between diseases but can also reduce the labor of animal keepers. However, the purity, safety, efficiency, duration of the effects and production costs of these kinds of vaccines have become problems that should be of great concern. Therefore, the combined vaccination of some single-disease vaccines has become a good strategy to solve these problems in the development of combined vaccines.

In the present study, the estimated three-dimensional structure of the chimeric protein rP97R1P46P42 was predicted (Fig. 1). All protein subunits of the combined vaccination component were expressed in prokaryotic cells, purified, and identified (Figs. 2 and 3). PCV2 Cap VLPs were observed (Fig. 4). Three polyclonal antibodies were developed and purified. All subunits of the chimeric protein and VLPs were recognized by specific antibodies (Fig. 5), which indicates that the components of the vaccine maintained the original antigenic epitopes.

In a lymphocyte proliferation assay, result indicates that combined vaccination could induce cellular immune responses in mice (Fig. 6). The total IgG antibody levels reflect the level of the systemic humoral immune response. Sera from the Pro group had good reactivity with all four antigens, which maintained increasing trends during the mouse immunization phase (Fig. 7). Results of IgG isotypes levels suggest that the combined vaccination induced a mixed Th1/Th2-type response in mice, which show as strong humoral immunity against antigens from Mhp and a strong cellular immune effect against PCV2 in mice (Fig. 8). The predominant production of humoral immunity induced by the rP97R1P46P42 chimera is consistent with research by de Oliveira et al. (2017), but the level of the cellular immune effect is lower than that in their studies. This discrepancy may be due to the different types of adjuvant we used. Studies have shown that oil adjuvants can induce a higher IgG2 immune response [31]. Freund’s adjuvant as an oil adjuvant is widely used in human and animal preliminary immune experiments, and has played a good adjuvant role in the results of other researchers [32–39]. However, it seems that his effect on the production of chimeric antigen-specific antibody is not good. Analyses of lymphocyte proliferation levels and total IgG and IgG isotype levels show that lymphocytes or sera from the Mhp CV group did not react with all the antigens. This can be ascribes to the commercial Mhp vaccine strain does not produce these antigens or expressed them at low levels, which was similar to the results of other researchers [4, 5, 15, 23, 40]. For the detection of Mhp strain 168 and PCV2 strain ZJ/C extracts as antigens, serum showed good reactivity with native proteins from these two strains (Fig. 9). In this study, Cap VLPs played an important role in combined vaccination, which manifested in better induction of humoral and cellular immunity against Cap than that induced by the PCV2 commercial vaccine.

In piglet immunization, production of antibody against four antigens indicates that the combined vaccination of two proteins could also induce a strong humoral immune response in piglets. IFN-γ is a macrophage-activating factor, which is a typical cytokine of Th1-type cells. It can enhance the activity of Th1 cells and promote cellular immune function. IL-4 is a cytokine secreted by Th2-type cells that can promote the proliferation of B cells. The high level of IFN-γ and IL-4 (Fig. 11) indicates that combined vaccination could induce a mixed Th1/Th2-type responses in pigs.

Commercial vaccines are generally produced by attenuating or inactivating field strains; however, due to the different kinships of different strains, there may be great differences in the expression levels of important antigens. Therefore, in practical applications, because of the different environments of pig farms, commercial vaccines may not always have a good protective effect against local field strains. In contrast, the vaccine produced by the specific antigens of pathogens using a genetic engineering method keeps a good immune effect [41, 42]. Despite the antigen selection of the Mhp chimera in this study is based on the results of de Oliveira et al. (2017), we analyzed the function of the four antigens they selected, screened out three of them, and further evaluated their immunogenicity in piglets. Although chimeras developed with more antigen components may be able to induce more comprehensive immunity, the results showed that the humoral immune response induced by the chimera we developed could be more balanced through serum analysis of IgG levels against subunit antigens. The formation of a chimera from fewer subunits may be more beneficial to the exposure of these subunit epitopes. Although the number of samples in pig immunization experiments is limited, it is enough to prove that combined immunization can induce humoral and cellular immune effects in piglets. It also shows good compatibility between the two components, which provides a theoretical basis for a large number of challenge experiments in next step.

## Conclusions

The combined vaccination of two proteins, which were both developed by genetic engineering method, showed good antigenicity and immunogenicity against specific antigens in mice and piglets. The present study demonstrates that: immunization of proteins from Mhp and PCV2 can induce humoral and cellular immune effects in mice and pigs; (2) chimeric of Mhp antigens have good component compatibility with Cap VLPs of PCV2. These results will provide a theoretical basis for using Mhp and PCV2 proteins to protect pigs against MPS and PCVD/PCVAD. This experiment provides a basis for further challenge experiments. Further study will focus on the combined vaccination of these two proteins on pigs in a double-challenge infection of Mhp and PCV2. The protective effect will be analyzed on the clinical levels such as the severity of lung lesions, the level of PCV2 viraemia and the average daily gain of infected pigs in experiment and field environment.

## Methods

### Selection of coding sequences and gene design

According to the previous research results regarding amino acid sequence selection of antigens, coding DNA sequences (CDSs) and protein sequences for P97R1 (MHP168_110), P46 (MHP168_522), and P42 (MHP168_069) from Mhp strain 168 (GenBank ID: CP002274.1) were used as references to design a chimeric protein antigen [4]. Cap (lacking the nuclear localization signal of 41 amino acid residues) from the PCV2 isolate ShanDong3–2016 (KY656098.1) was used as the other antigen (Table 1) [43]. A flexible linker GGSG (GlyGlySerGly) was inserted between each chimeric subunit protein of Mhp to enable the proper folding of the protein. The chimeric gene of Mhp was named *rp97R1p46p42*. The three-dimensional structure of the chimeric protein rP97R1P46P42 was predicted using the I-TASSER online software (https://zhanglab.ccmb.med.umich.edu/I-TASSER/) [44].

### Cloning, expression, purification, and identification of recombinant proteins

The *rp97R1*, *rp46*, and *rp42* (both were amplified from *rp97R1p46p42*), *rp97R1p46p42*, and *cap* genes were cloned into the pET32a vector (Novagen, USA) as described previously [40]. The recombinant plasmids were sequenced by Shanghai Bioengineering Co., Ltd. The *E. coli* DH5α and BL21 (DE3) strains (Invitrogen, USA) were used for plasmid DNA amplification and recombinant protein expression, respectively.

The pET32a vector expressed protein, rP97R1, rP46, rP42, rP97R1P46P42, and Cap proteins were both expressed and purified using a previously described method [45]. Proteins were identified by SDS-PAGE.

### Purification and observation of cap VLPs

To verify the assemblage of the PCV2 Cap VLP, the Cap protein was further purified by sucrose density gradient ultracentrifugation. Briefly, the proteins were pelleted by centrifugation at 35,000 rpm for 4 h at 4 °C (SW41 Rotor, Beckman, USA), followed by transferring to a 20–60% (w/v) discontinuous sucrose gradient and centrifugation at 35000 rpm for 2 h at 4 °C for purification of VLPs. The pellet was finally removed from sucrose by ultracentrifugation and resuspended in PBS. Purified VLPs were adsorbed onto a copper grid, stained with 3% phosphotungstic acid, and observed by transmission electron microscopy (TEM, Hitachi 7650, Japan).

### Preparation of polyclonal antibodies

All procedures for handling the animals used in this study were reviewed and approved by the College of Life Sciences of Zhejiang Sci-Tech University and performed in an ethical and humane manner under veterinary supervision. For the production of polyclonal anti-rP97R1, anti-rP46, and anti-rP42 antibodies, three 2.5-kg female New Zealand White rabbits (purchased from the Animal Center of Zhejiang Chinese Medical University) were subcutaneously immunized with 1 mg of rP97R1, rP46, and rP42 at 10-day interval. Proteins were administered with complete Freund’s adjuvant (Sigma, USA) on day 0 and were injected with incomplete Freund’s adjuvant (Sigma, USA) on day 10, 20 and 30 post the first immunization. At day 37 post the first immunization, under the anesthesia of rabbits, blood was obtained by cardiac puncture and serum was separated. After that, the rabbits were euthanized by administering sodium pentobarbital (60 mg/kg) intravenously, while the death was verified by the lack of cardiac pulse and fixed and dilated pupils prior to carcass disposal. Polyclonal antibodies were purified by Protein A column (Beyotime, China) from serum. After euthanasia, the rabbit bodies were handed over to the DADI WEIKANG Company for harmless treatment immediately.

### Western blotting analysis

Western blots were performed in this study using a previously described method [15]. The rP97R1, rP46, rP42, rP97R1P46P42, and Cap proteins were further identified by Western blotting using a monoclonal anti-His antibody (1:5000; Beyotime, China). To evaluate the antigenicity of rP97R1P46P42 expressed in *E. coli*, polyclonal anti-rP97R1, anti-rP46, and anti-rP42 antibodies (1:1000) obtained above were used in the Western blotting, and anti-Cap antibody (1:5000; Bioss, China) was used to evaluate the antigenicity of Cap VLPs.

### Cell and virus culture and preparation of mycoplasma

The porcine kidney cell line (PK-15, kindly provided by Ebvac, China), free of PCV2, was maintained in Dulbecco’s modified Eagle’s medium (DMEM; Gibco, USA) supplemented with 10% (v/v) heat-inactivated fetal bovine serum (FBS; Gibco, USA), 100 U/mL penicillin, and 100 μg/mL streptomycin. The ZJ/C strain of PCV2 was provided by Ebvac and was propagated and titrated in PK-15 cells following standard protocols [46]. The 168 strain of Mhp was obtained from Zhibining® (Nanjing Tech-Bank Bio-Industry, China) and propagated in Friis medium using a previously described method [47].

### Immunization of mice

A total of thirty female 6–8-week-old BALB/c mice (Cleanliness: SPF; purchased from the Animal Center of Zhejiang Chinese Medical University) were kept in individually ventilated cage (IVC) system. All breeding, operation and care of this experiment were carried out in accordance with the “Guidelines for the Management and Use of Experimental Animals”. During the experiment, humane care was given in accordance with the 3R principle of experimental animals, and was approved by the Experimental Animal Welfare Ethics Committee of Zhejiang Sci-Tech University.

Mice were randomly divided into five groups and six animals per group: PBS group, PBS + Freund’s adjuvant;pET32a vector protein (VP) group, VP+ Freund’s adjuvant; Mhp commercial vaccine (Mhp CV) group, RespiSure® ONE (Harbin Pharmaceutical Group, China); PCV2 commercial vaccine (PCV2 CV) group, Yuankexin® (Pulike, China); Combined vaccination (Pro) group, rP97R1P46P42 + Cap VLPs + Freund’s adjuvant. A 14-day interval subcutaneous injection was used for all the groups of mice. The mice in PBS group were immunized with 100 μL PBS, the VP group mice were immunized with 50 μg pET32a vector-expressed protein, the Pro group mice were immunized with a mixture of 50 μg of rP97R1P46P42 and 50 μg of Cap VLPs, the Mhp CV group mice were immunized with 100 μL of RespiSure® ONE and the PCV2 CV group mice were immunized with 100 μL of Yuankexin®. PBS and proteins were administered in complete Freund’s adjuvant (Sigma, USA) on day 0 and in incomplete Freund’s adjuvant on days 14 and 28. The protein and complete Freund’s adjuvant (CFA) were mixed at 1:1 ration. According to the instruction of CFA provided by Sigma Company and Guidelines for the Use of Adjuvants in Research: Special Emphasis on Freund’s Adjuvant (Animal Research Advisory Committee Guidelines; NIH Office of Instramural Research Office of Animal Care and Use), and usages in other reports [16, 48, 49], the concentration of *Mycobacterium tuberculosis* is 1 mg/mL, and the final concentration of *Mycobacterium tuberculosis* is 0.5 mg/mL in the emulsion. Serum samples were obtained from the retro-orbital sinus 0, 14, 28, 35, and 42 days after the first immunization (DAI). Three mice of each group were euthanized by administering sodium pentobarbital (60 mg/kg) intraperitoneally, while the death was verified by the lack of cardiac pulse and fixed and dilated pupils prior to carcass disposal. After euthanasia, the spleen was separated for a lymphocyte proliferation assay at 35 and 42 DAI. All of the mouse bodies was temporarily stored at − 80 °C and handed over to DADI WEIKANG Company for harmless treatment at the end of the whole experiment period.

### Immunization of piglets

To determine the immunogenicity of the combined vaccination in pigs, six 4-week-old male piglets with the weight of 7–8 kg from a Mhp- and PCV2-free herd (Cleanliness: clean; purchased from the Animal Center of Zhejiang Chinese Medical University), were housed in the IVC system. All breeding, operation and care of this experiment were carried out in accordance with the “Guidelines for the Management and Use of Experimental Animals”. During the experiment, humane care was given in accordance with the 3R principle of experimental animals, and was approved by the Experimental Animal Welfare Ethics Committee of Zhejiang Sci-Tech University. After the entire experiment period is over, all the piglets were euthanized by intravenous administration of sodium pentobarbital (90 mg/kg), while the death was verified by the lack of cardiac pulse and fixed and dilated pupils prior to carcass disposal. After euthanasia, the piglet bodies were handed over to the DADI WEIKANG Company for harmless treatment immediately.

Pigs were randomly divided into two groups, three animals for each group: negative control (pET32a vector-expressed protein, VP) group, intramuscularly administered with 1 mg of pET32a vector protein per animal at 0 and 14 DAI; combined vaccination (Pro) group, intramuscularly administered with 1 mg of rP97R1P46P42 and 1 mg of Cap VLPs per animal twice with two-week interval between vaccinations. Proteins were administered with complete Freund’s adjuvant on day 0 and in incomplete Freund’s adjuvant on day 14. Serum samples were obtained from veins at 0, 14 and 28 DAI.

### Lymphocyte proliferation assay

Splenic lymphocytes were isolated from the spleens of immunized mice with lymphocyte separation medium (DAKEWEI, China) according to the manufacturer’s instruction, and lymphocyte proliferation assays were performed as described previously [44]. The lymphocytes were seeded into the plates at 5 × 10^5^ cells/well. 2 μg of rP97R1, 2 μg of rP46, 2 μg of rP42, and 2 μg of Cap were mixed and added into each well as a stimulator. RPMI-1640 medium was used as a negative control, and Concanavalin A (5 μg/mL; Sigma, USA) was used as a positive control. Each splenic lymphocyte sample was plated in triplicate. The proliferative activity was measured by a standard MTT method. The optical density (OD) was determined at a wavelength of 490 nm, and the stimulation index (SI) was calculated as follows: SI = mean OD of antigen-stimulated cells (rP97R1, rP46, rP42, and Cap added)/mean OD of unstimulated cells (RPMI-1640 added).

### Enzyme-linked immunesorbent assays (ELISAs)

The antibody titers were determined by a modified ELISA as previously described [50]. Briefly, 96-well plates were coated with 100 ng of rP97R1, rP46 or rP42 for each well. Then, the proteins were incubated with serially diluted sera. HRP-conjugated goat anti-rabbit IgG (1:1000; Beyotime, China) was used as a secondary antibody. The absorbance was measured at 450 nm in an ELISA Microplate Reader (Biotek, USA).

The specificity of the antibodies elicited by immunization with vaccine formulations was determined by ELISA. Microtiter plates were coated with each chimeric subunit protein or Cap protein (rP97R1, rP46, rP42 or Cap: 100 ng/well). To determine the level of the total IgG, the antigen-coated plates were followed by incubation with diluted serum samples collected from the immunized mice or pigs on 0, 14, 21, 35, and 42 DAI and HRP-conjugated goat anti-mouse IgG (1:1000; Beyotime, China). To determine the levels of IgG isotypes, the antigen-coated plates were followed by incubating with diluted serum samples from 0 and 42 DAI and HRP-conjugated goat anti-mouse IgG1 and IgG2a (1:30,000; Abcam, USA), respectively. To intuitively reflect the difference in antibody levels among the five groups. The serum samples were diluted at 1: 51,200 for rP97R1 and rP42 and 1: 25,600 for rP46 and Cap. The remaining steps were similar to those described above. Each serum sample was plated in triplicate.

An ELISA was performed with sera from the mice immunized with vaccine formulations to verify whether the antibodies induced by these antigens were able to recognize native proteins from the field strains of virus and Mycoplasma, as previously described [45]. To this end1 μg of crude extract of Mhp strain 168 or PCV2 strain PC/J was coated to each well of microtiter plates. The plates were incubated at 4 °C overnight, then incubated at − 80 °C for 2 h and thawed at room temperature for 30 min. Then, the same procedure was carried out as described above. Each serum sample was plated in triplicate.

The analysis of antibody levels in the serum of immunized piglets was similar to that of IgG levels in the serum of immunized mice. rP97R1, rP46, rP42 and Cap were coated at 100 ng/well followed by incubating with serum samples (1:400) of piglets from 0, 14 and 28 DAI and HRP-conjugated rabbit anti-pig IgG (1:5000; Bioss, China). Each serum sample was plated in triplicate.

The cellular response induced in pigs was analyzed by the detection of the IFN-γ and IL-4 cytokines in the sera of immunized piglets, which was performed using commercial mouse cytokine double antibody sandwich ELISA kits (FEIYA Biological Technology, China) according to the manufacturer’s instructions.

### Statistical analysis

Statistical analysis was performed using GraphPad Prism® Version 6 for Windows (GraphPad Software Inc., San Diego, CA, USA). Data were expressed as the mean ± SD. Analysis of variance (ANOVA) followed by Bonferroni post-test were used to compare the immune responses between the different groups. Data were considered significantly different when *P* was < 0.05.

## Supplementary information

**Additional file 1.**

**Additional file 2.**

**Additional file 3.**

**Additional file 4.**

**Additional file 5.**

**Additional file 6.**

**Additional file 7.**

**Additional file 8.**

**Additional file 9.**

## Data Availability

All data generated or analysed during this study are included in this published article and its supplementary information files. The datasets used and/or analysed during the current study available from the corresponding author on reasonable request.

## Abbreviations

Mhp: *Mycoplasma hyopneumoniae*
PCV2: Porcine circovirus type 2
Cap: Capsid protein of PCV2
MPS: Mycoplasma pneumonia of swine
PEP: Porcine enzootic pneumonia
EP: Enzootic pneumonia
PCVDs/PCVADs: porcine circovirus diseases and porcine circovirus-associated diseases
PMWS: Postweaning multisystemic wasting syndrome
PDNS: Porcine dermatitis nephritic syndrome
CDS: Coding DNA sequences
PCR: Polymerase chain reaction
*E. coli*: *Escherichia coli*
ELISA: Enzyme-linked immunosorbent assay
PK-15: Porcine kidney cell line
DMEM: Dulbecco’s modified Eagle’s medium
FBS: Fetal bovine serum
DAI: Days after first immunization
MTT: 3-(4,5-dimethylthiazol-2-yl) 2,5-diphenyltetrazolium bromide
OD: Optical density
I: Stimulation index
TEM: Transmission electron microscopy
