# Supplementary figures and images for: Immune responses induced by a combined vaccination with a recombinant chimera of Mycoplasma hyopneumoniae antigens and capsid virus-like particles of porcine circovirus type 2
Source: BMC Vet Res. 2020 Sep 16;16:342. doi: 10.1186/s12917-020-02560-8 (PMC7493066; doi:10.1186/s12917-020-02560-8)

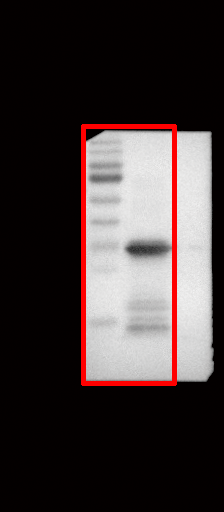

Supplement: Supplementary file 1 — Additional file 1. [file 12917_2020_2560_MOESM1_ESM.tif]

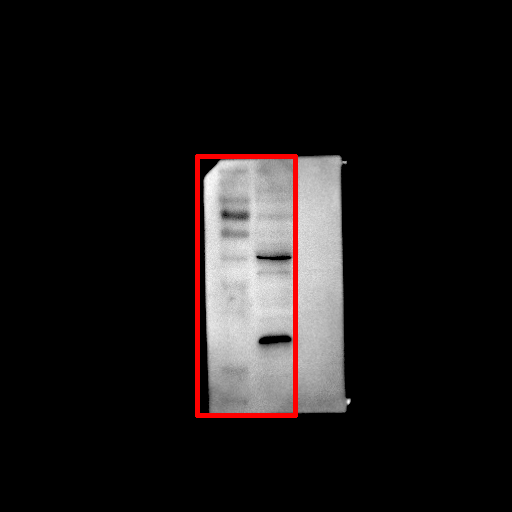

Supplement: Supplementary file 2 — Additional file 2. [file 12917_2020_2560_MOESM2_ESM.tif]

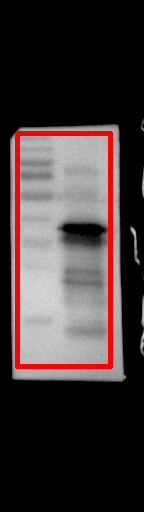

Supplement: Supplementary file 3 — Additional file 3. [file 12917_2020_2560_MOESM3_ESM.tif]

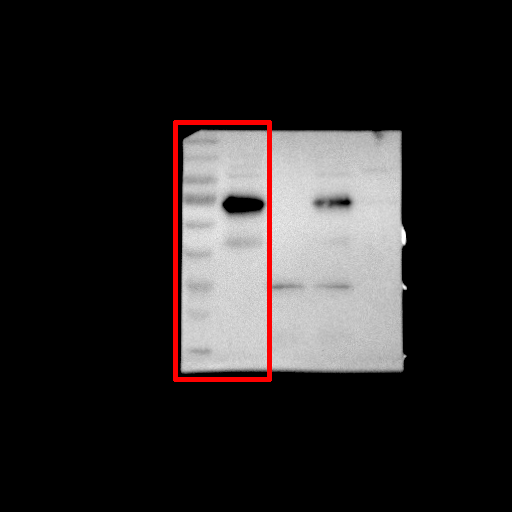

Supplement: Supplementary file 4 — Additional file 4. [file 12917_2020_2560_MOESM4_ESM.tif]

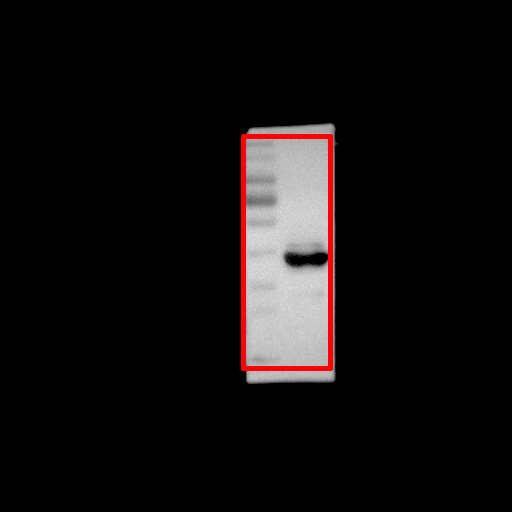

Supplement: Supplementary file 5 — Additional file 5. [file 12917_2020_2560_MOESM5_ESM.tif]

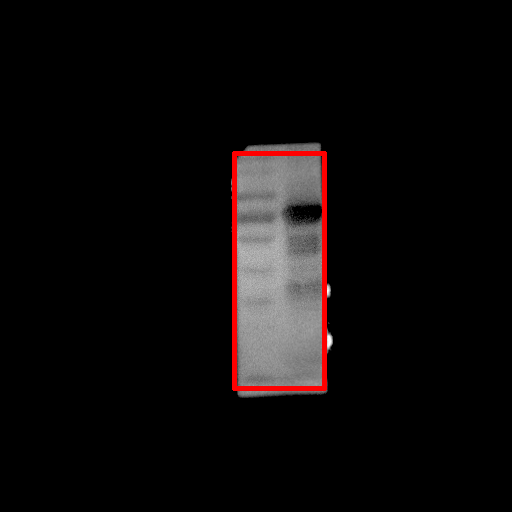

Supplement: Supplementary file 6 — Additional file 6. [file 12917_2020_2560_MOESM6_ESM.tif]

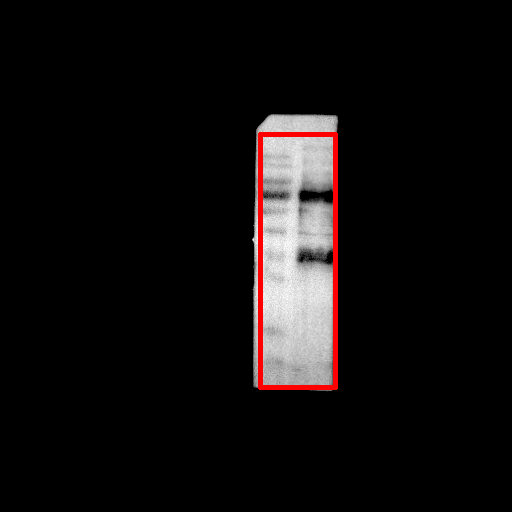

Supplement: Supplementary file 7 — Additional file 7. [file 12917_2020_2560_MOESM7_ESM.tif]

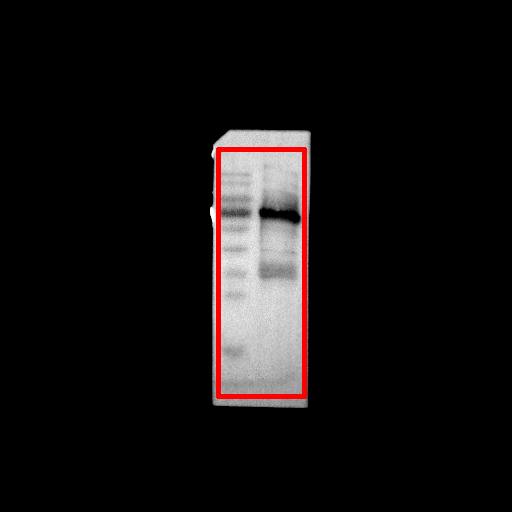

Supplement: Supplementary file 8 — Additional file 8. [file 12917_2020_2560_MOESM8_ESM.tif]

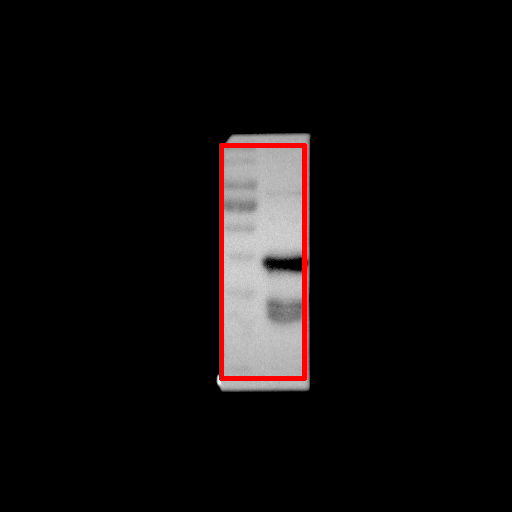

Supplement: Supplementary file 9 — Additional file 9. [file 12917_2020_2560_MOESM9_ESM.tif]
